# Short-term outcomes of the PreserFlo MicroShunt in Japanese patients with exfoliation glaucoma: a comparison with primary open-angle glaucoma using propensity score matching

**DOI:** 10.1007/s10384-025-01265-5

**Published:** 2025-08-26

**Authors:** Teruki Yamae, Rei Sakata, Haruyuki Suzuki, Yurika Aoyama, Hotaka Nemoto, Hitomi Saito, Megumi Honjo, Shiroaki Shirato, Makoto Aihara

**Affiliations:** 1 Department of Ophthalmology, Graduate of Medicine and Faculty of Medicine, The University of Tokyo Hospital, 7-3-1 Hongo, Bunkyo-ku, Tokyo, 113-8655 Japan; 2 Yotsuya Shirato Eye Clinic, Tokyo, Japan

**Keywords:** PreserFlo MicroShunt, Intraocular pressure, Exfoliation glaucoma, Primary open-angle glaucoma, Propensity score matching

## Abstract

**Purpose:**

This study evaluated the efficacy and safety of initially implanted PreserFlo MicroShunt (PMS) in Japanese patients with exfoliation glaucoma (XFG). Using propensity score matching, intraocular pressure (IOP) control rates were compared between patients with XFG and primary open-angle glaucoma (POAG).

**Study design:**

Retrospective observational study.

**Methods:**

This study reviewed 31 eyes of 31 patients with XFG who underwent initial PMS implantation with mitomycin C. IOP, medication scores, and corneal endothelial cell density (CECD) were assessed preoperatively and at up to 6 months postoperatively. Kaplan–Meier analysis was used to estimate the 6-month survival rate, defined as an IOP reduction of > 20% from baseline and an IOP < 15 mmHg. The incidences of needling, reoperation, and complications were also assessed. IOP control was compared between XFG and propensity-score-matched POAG patients using the log-rank test.

**Results:**

At 6 months, the mean IOP had decreased significantly, from 22.3 ± 6.6 to 14.7 ± 6.6 mmHg, and the medication score had declined from 4.5 to 1.4. CECD decreased from 2127 to 1902 cells/mm^2^, although this was not statistically significant. The complete success rate (without any glaucoma medications or intervention) was 48%. Postoperative complications included anterior chamber hemorrhage and choroidal detachment. Needling was performed in nine eyes (29.0%), and additional surgery was performed in five eyes (16.1%). Compared to POAG patients (11.9 mmHg), XFG patients had higher postoperative IOP (14.8 mmHg) and higher medication scores (0.5 vs 1.4, *p* = 0.04) and a lower success rate (62.2% vs 41.7%).

**Conclusions:**

PMS in Japanese patients with XFG resulted in a significant IOP reduction over 6 months, with a relatively favorable safety profile. However, its efficacy was slightly inferior to that in POAG, implying potential differences in PMS responsiveness between glaucoma subtypes.

## Introduction

Compared to primary open-angle glaucoma (POAG), exfoliation glaucoma (XFG), a subtype of secondary glaucoma [1], is characterized by a higher prevalence in elderly patients, an elevated intraocular pressure (IOP) with larger IOP fluctuations, and more rapid disease progression [2–4]. Consequently, achieving adequate IOP control with topical medications alone is often challenging, frequently necessitating surgical intervention. Because XFG is associated with increased resistance to aqueous humor outflow [5], outflow reconstruction surgery (i.e., minimally invasive glaucoma surgery; MIGS) is theoretically effective. However, the postoperative IOP in cases after MIGS typically stabilizes in the mid-teens [6], which may be insufficient for XFG patients in the middle to late stages of glaucoma [7].

Trabeculectomy is the only surgical procedure that can reduce IOP to a single-digit range, making it effective for severe cases. However, it demands complex postoperative management and carries a risk of adverse effects on vision, necessitating careful consideration of the patient’s age, systemic health, and the condition of the opposite eye. The long-term success rates of trabeculectomy are reported to be lower in XFG than in POAG [8, 9]. This difference is thought to result from the more pronounced postoperative inflammation in XFG [10, 11], which may lead to conjunctival adhesion and reduced functionality of the filtering bleb. However, many confounding factors should be considered to compare the surgical outcomes of trabeculectomy among eyes with different subtypes, in light of the surgical differences among surgeons, and postoperative management of the bleb.

The PreserFlo MicroShunt (PMS) has emerged as a promising new glaucoma filtration surgery treatment. This involves the insertion of a poly(styrene-block-isobutylene-block-styrene) tube between the anterior chamber and subconjunctival tissue, creating a new aqueous humor outflow path through a minimally invasive filtration procedure. The PMS eliminates the need to create a scleral flap or remove peripheral corneal/scleral tissue, thereby reducing intra- and postoperative invasiveness. Compared to trabeculectomy, the PMS has less variation in the surgical steps and postoperative IOP management. Thus, the PMS is useful for evaluating the surgical outcomes of glaucoma subtypes or risk assessment. The PMS is already widely used internationally, and in patients with a preoperative IOP of around 20 mmHg on several medications, IOP is reported to stabilize in the low teens postoperatively [12–25]. A study of Japanese patients with POAG shows significant IOP reductions to around 10 mmHg within 6 months after surgery, without major vision-threatening complications [26].

The risk factors for postoperative IOP control failure after PMS insertion include pigmentary glaucoma, primary angle-closure glaucoma, non-Caucasian ethnicity, low-dose intraoperative mitomycin C (MMC), concurrent cataract surgery, and XFG [27]. Although international studies indicate that the PMS effectively reduces IOP and decreases the need for glaucoma medications in XFG [17, 28], data on Japanese (Asian) patients with XFG remain limited [29]. Therefore, assessing the efficacy and safety of the PMS in Japanese patients with XFG is important clinically.

This study compared the outcomes and safety of the PMS in Japanese patients with XFG and POAG using propensity score matching to align the preoperative conditions. The findings should provide valuable insights into the effectiveness of the PMS in patients with XFG and clarify its role in clinical practice.

## Methods

This retrospective cohort study analyzed the medical records of patients with XFG who underwent their first PMS surgery with MMC between October 2022 and December 2023 at the University of Tokyo Hospital or the Yotsuya Shirato Eye Clinic. This study adhered to the principles of the Declaration of Helsinki and was approved by the institutional ethics committee (no. 2217). Informed consent was obtained from all participants. Only the first operated eye was included in the analysis for patients who underwent bilateral surgery, ensuring one eye per patient.

### Inclusion criteria

Eyes with visible exfoliation material on anterior segment examination and evidence of glaucomatous optic neuropathy; phakic and pseudophakic eyes were eligible; no restrictions were imposed on age, sex, or the stage of glaucoma; patients with a postoperative follow-up period of at least 6 months; and eyes with a history of previous eye surgery that did not involve conjunctival manipulation (cataract surgery alone, *ab interno* outflow reconstruction surgery alone, selective laser trabeculoplasty, or laser iridotomy).

### Exclusion criteria

Eyes with a history of surgery or procedures that can lead to conjunctival scarring, including trabeculectomy, glaucoma implant surgery, vitrectomy, retinal detachment surgery, or transscleral cyclophotocoagulation.

### Surgical procedure

Nine surgeons participated in this study. Sub-Tenon’s anesthesia with 2% lidocaine was administered. The conjunctiva and Tenon’s capsule were incised and dissected at the fornix base at either superonasal or superotemporal quadrant at the discretion of the operating surgeon. A sponge with MMC (0.04% for 3 minutes or 0.05% for 1.5 minutes) was applied to the surgical site, followed by thorough irrigation with a balanced salt solution. For cases involving combined cataract surgery, lens reconstruction through a corneal incision was performed at this stage. A scleral tunnel was created 3 mm posterior to the limbus using a double-step knife [30], and the PMS was inserted. Aqueous humor outflow from the distal end of the device was confirmed before suturing Tenon’s capsule and the conjunctiva with 10-0 nylon (MANI, Inc.,). The procedure was completed by applying betamethasone valerate ointment and ofloxacin ophthalmic ointment.

### Postoperative care

Patients were prescribed topical gatifloxacin and betamethasone sodium phosphate (0.1%) eye drops four times daily. For cases involving combined cataract surgery, bromfenac eye drops were also prescribed twice daily. These medications were tapered and discontinued at the surgeon’s discretion, except for the betamethasone eye drops, which were continued for 6 months postoperatively. In cases of postoperative IOP elevation or complications, additional glaucoma medications, needling, or repeat surgery were performed at the surgeon’s judgment.

### Examinations and evaluation parameters

Data were collected at specific time points based on follow-up observations up to 6 months postoperatively. The measurement time points were preoperatively (baseline), 1 week (± 2 days), 1 month (± 7 days), 3 months (± 28 days), and 6 months (± 56 days) after surgery.

**IOP measurement**: IOP was measured using a Goldmann applanation tonometer under topical anesthesia in the sitting position.

**Glaucoma medication score**: The score was calculated as follows: one point for each active component in monotherapy eye drops, two points for combination eye drops, and one point for oral acetazolamide use.

**Corneal endothelial cell density (CECD)**: CECD at the central cornea was measured using a specular microscope (Tomey) at baseline and at 3 and 6 months postoperatively.

**Other examinations**: Slit-lamp and fundus examinations were conducted at each follow-up visit.

### Evaluation parameters and statistical analysis

The temporal changes in IOP, glaucoma medication scores, and CECD were analyzed using a mixed-effects model to evaluate the significance of changes from baseline at each time point. Postoperative interventions and complications were thoroughly reviewed based on medical records.

To evaluate the treatment efficacy, the following criteria were used:

1. Complete success: an IOP reduction of ≥ 20% from preoperative IOP, maintained within 6 to 14 mmHg without additional interventions or glaucoma medications.
2. Qualified success: The above criteria were met but required postoperative needling or glaucoma medications.

Exceptions were made for IOP values, medication use, or needling occurring within the first postoperative month. Survival rates were analyzed using the Kaplan–Meier method, with failure defined as two consecutive IOP measurements outside the success criteria or requiring additional interventions or repeat surgery.

For comparison, patients with POAG who underwent PMS during the same period were included. Propensity score matching was performed to adjust for the confounding factors: age, sex, combined cataract surgery, preoperative IOP, and topical medication scores [31]. The caliper for matching was set at 0.2. The inclusion criteria for POAG were: presence of a normal open angle, glaucomatous disc and peripapillary retinal changes corresponding to the visual field defect, visual field defect based on the Anderson and Patella criteria [32], and eyes with phakic or intraocular lens (IOL). The exclusion criteria were: the presence of angle-closure glaucoma, exfoliation glaucoma, secondary glaucoma, or childhood glaucoma; history of any glaucoma surgery including laser treatment, pars plana vitrectomy, and buckling surgery with a conjunctival incision; and history of uveitis, steroid use, trauma, ocular infections, inflammatory diseases, corneal diseases, or retinal diseases.

Pre- and postoperative IOP and medication scores were compared using the Student’s* t*-test. Fisher’s exact test was used to assess the differences in postoperative success rates and complication frequencies. A paired t-test was performed for IOP and medication score after propensity score matching. The log-rank test was used to evaluate the differences in survival rates between the glaucoma subtypes. The statistical analyses were performed using JMP Pro ver. 17.0 (SAS) and R version 3.4.3 (The Foundation for Statistical Computing), with statistical significance defined as *p* < 0.05.

## Results

The analysis included 31 XFG cases (31 eyes). The patient background is shown in Table 1. The patients’ mean age (± standard deviation) was 77.5 ± 5.8 years, with one eye being phakic and the remaining 30 eyes having IOL implants. Eleven eyes had a history of reconstruction surgery and four had a history of glaucoma laser treatment. The preoperative mean IOP was 22.3 ± 6.6 mmHg, glaucoma medication score was 4.5 ± 1.0, and the CECD was 2127 ± 419.9 cells/mm^2^.

**Table 1:** Patients’ characteristics

	*n* = 31
Male/Female (*n*)	16/15
Age (years)	77.5 ± 5.8 (64–86)
Laterality (right / left) (eye)	14/17
Lens (Phakia / Pseudophakic) (eye)	1/30
Combined use with phacoemulsification (eye)	1
History of MIGS / SLT or MPCPC (eye)	11/4
Visual field mean deviation (dB)	30-2: -15.5 ± 1.3dB (3 eyes) 10-2: -17.8 ± 7.2dB (21 eyes) Other (7 eyes)
Preoperative IOP (mmHg)	22.3 ± 6.6 (10–36)
Preoperative glaucoma medication score	4.5 ± 1.0 (3–6)
Corneal endothelial cell density (cells/mm^2^)	2127 ± 420 (1368–2705)

Data are shown mean ± standard deviation (range)

*n* number, *dB* decibel, *IOP* intraocular pressure, *MIGS* minimally invasive glaucoma surgery, *SLT* selective laser trabeculoplasty, *MPCPC* micropulse cyclophotocoagulation

**Fig. 1:**
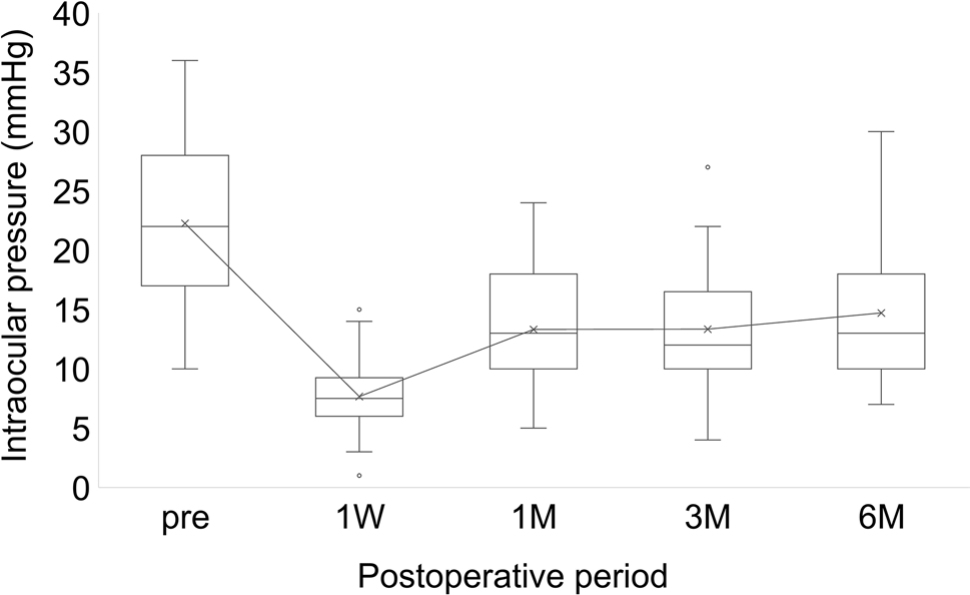
Changes in mean intraocular pressure (IOP) before and after insertion of a PreserFlo MicroShunt. The vertical axis indicates IOP (mmHg), while the horizontal axis represents time. The whiskers of the box plot denote the range of data within 1.5 times the interquartile range (IQR) from the first and third quartiles. The bar is a line drawn between the maximum and minimum values. A significant reduction in IOP was observed at all postoperative time points (*p* < 0.001) as determined by the Student’s t-test

**Fig. 2:**
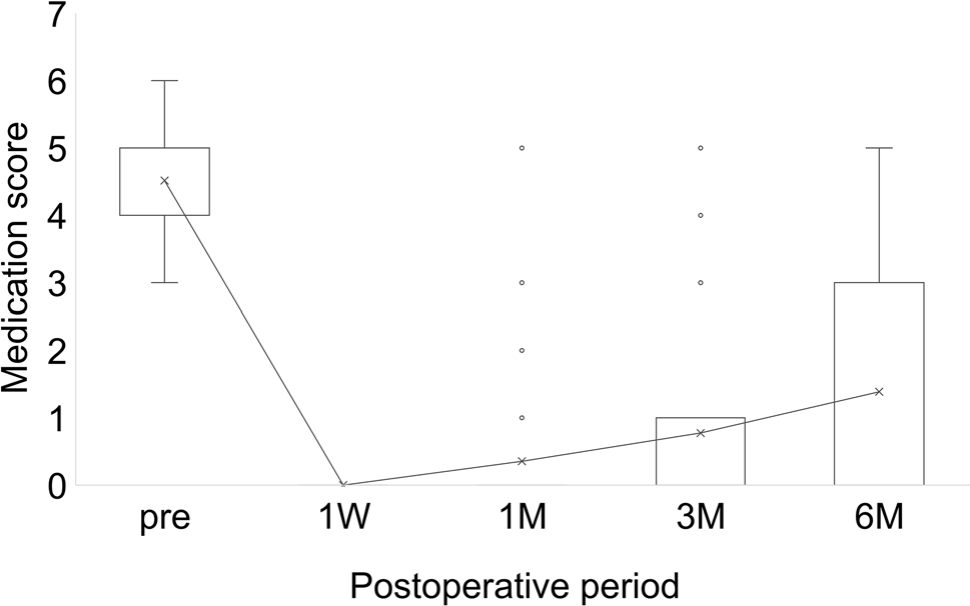
Changes in mean glaucoma medication scores before and after insertion of a PreserFlo MicroShunt. The vertical axis represents Glaucoma medication scores, while the horizontal axis indicates time. The whiskers of the box plot denote the range of data within 1.5 times the interquartile range (IQR) from the first and third quartiles. The bar is a line drawn between the maximum and minimum values. A significant reduction was observed at all postoperative time points (*p* < 0.001) as determined by the Student’s t-test

The mean IOP was significantly (*p* < 0.001) lower than preoperatively (22.3 ± 6.6 mmHg) at all time points: 1 week postoperatively (7.7 ± 3.2 mmHg), 1 month (13.3 ± 5.2 mmHg), 3 months (13.3 ± 5.2 mmHg), and 6 months (14.7 ± 6.1 mmHg) (Fig. 1). The glaucoma medication score was also significantly (*p* < 0.001) lower than preoperatively (4.5 ± 1.0) at all time points: 1 week postoperatively (0 ± 0), 1 month (0.4 ± 1.1), 3 months (0.8 ± 1.5), and 6 months (1.4 ± 1.8) (Fig. 2). The mean CECD showed no significant changes: preoperatively (2127.4 ± 427.7), 3 months (2024.7 ± 505.2), and 6 months (1902 ± 527.0) (*p* = 0.45 and 0.11, respectively). A decrease of more than 5% in CECD from the preoperative period to 6 months postoperatively was observed in 11 of 31 eyes (35.5%)

Needling was performed in nine eyes (29.0%), with an average time to treatment of 50.7 (range 5–127, median 37) days after surgery. Additional surgery was performed in five eyes (16.1%), including filtering bleb revision in three eyes (postoperative days 37, 65, and 90), vitreous surgery was performed in one eye (postoperative day 34), and sclerotomy was performed in one eye (postoperative day 15). Furthermore, four eyes required needling multiple times (Table 2). The most common postoperative complication was anterior chamber hemorrhage, observed in 10 eyes (32.3%). Choroidal detachment was noted in two eyes, and choroidal and vitreous hemorrhage were each observed in one eye (separate cases) (Table 2).

**Table 2:** Complications and postoperative interventions

	Eyes (%)	POD (days)
Hyphema	10 (32.3)	1
Hypotony (≤ 5mmHg)	6 (19.4)	1–7
Choroidal detachment	2 (6.5)	1
Suprachoroidal hemorrhage	1 (3.2)	3 (estimation)
Vitreous hemorrhage	1 (3.2)	9 (estimation)
*Interventions*		
Needling	9 (29.0)	Mean 50.7 (range 5–127; Median 37)
Additional surgery	5 (16.1)	
Bleb revision	3 (9.7)	37, 65, 90
PPV	1 (3.2)	34
Drainage sclerotomy	1 (3.2)	15

*POD* postoperative days, *PPV* pars plana vitrectomy

**Fig. 3:**
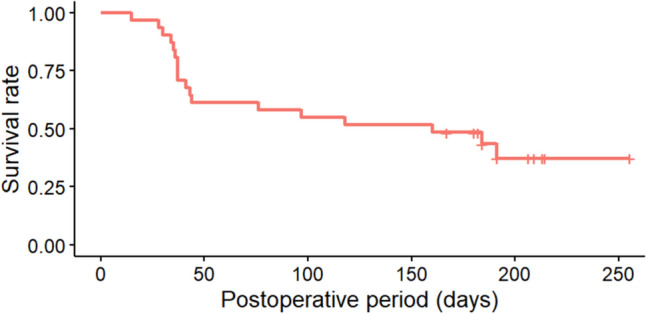
IOP survival rates based on the Kaplan–Meier method at up to 6 months after PreserFlo MicroShunt insertion (complete success). The vertical axis indicates the survival rate based on complete success, while the horizontal axis shows time (days). High intraocular pressure (IOP) values, medication use, or needling occurring within one month after surgery were considered acceptable for survival rate

**Fig. 4:**
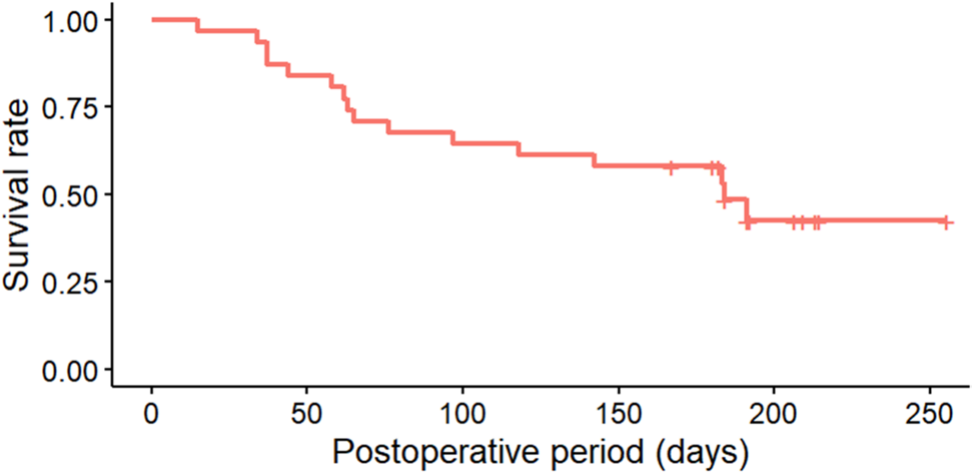
IOP survival rates based on the Kaplan–Meier method at up to 6 months after PreserFlo MicroShunt insertion (qualified success). The vertical axis indicates the survival rate based on qualified success, while the horizontal axis shows time (days). High intraocular pressure (IOP) values, medication use, or needling occurring within one month after surgery were considered acceptable for survival rate

At 6 months postoperatively, the survival rate (defined as ≥ 20% reduction in IOP from baseline, with an IOP within 6–14 mmHg) was 48.4% [95% confidence interval (95% CI), 30.8–66.0%] for complete success (Fig. 3) and 58.1% (95% CI, 40.7–75.4%) for qualified success (Fig. 4).

**Fig. 5:**
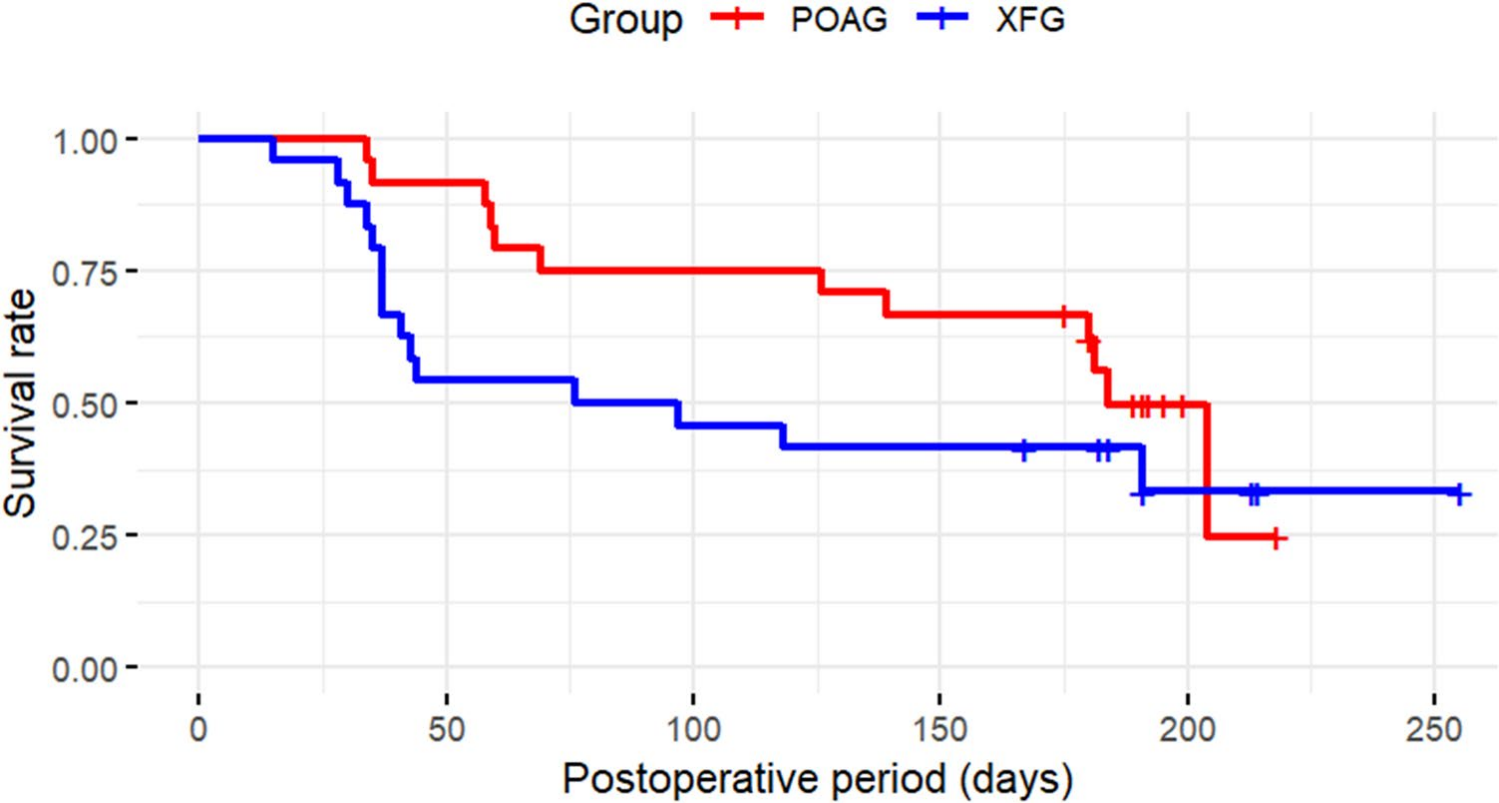
IOP survival rates based on the Kaplan–Meier method at up to 6 months after surgery in exfoliation glaucoma (XFG) and primary open-angle glaucoma (POAG) eyes after PreserFlo MicroShunt insertion (complete success). The vertical axis represents the survival rate based on complete success, while the horizontal axis indicates time (days). The blue line indicates XFG and the red line POAG. No significant difference was observed between the two groups (*p* = 0.20, log-rank test). High intraocular pressure (IOP) values, medication use, or needling occurring within one month after surgery were considered acceptable for survival rate

**Fig. 6:**
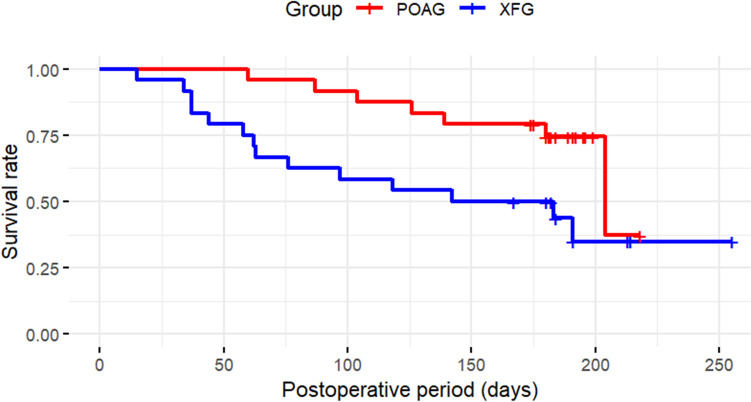
IOP survival rates based on the Kaplan–Meier method at up to 6 months after surgery in POAG and XFG eyes after PreserFlo MicroShunt insertion (qualified success). The vertical axis represents the survival rate based on qualified success, while the horizontal axis indicates time (days). The blue line indicates XFG, and the red line POAG. A significant difference was observed between the two groups (*p* = 0.03, log-rank test). High intraocular pressure (IOP) values, medication use, or needling occurring within one month after surgery were considered acceptable for survival rate

Propensity score matching was used to match the XFG patients in this study with POAG patients who underwent PMS during the same observation period. Ultimately, 24 eyes were included in each group of the study. At 6 months postoperatively, IOP was 11.9 ± 2.7 mmHg in the POAG group and 14.8 ± 6.1 mmHg in the XFG group, with significantly higher IOPs in the XFG group (*p* = 0.04). The glaucoma medication scores were 0.5 ± 0.9 in the POAG group and 1.4 ± 1.8 in the XFG group and were significantly higher in the XFG group (*p* = 0.04) (Table 3). IOP and medication score of the XFG and POAG groups are presented in Table 4. The survival rate for complete success was 62.2% (95% CI, 42.7–81.7%) in the POAG group and 41.7% (95% CI, 22.0–61.4%) in the XFG group, with no significant difference observed (log-rank test, *p* = 0.20) (Fig. 5). By contrast, for qualified success, the survival rate was 74.5% (95% CI, 56.8–92.2%) in the POAG group and 50.0% (95% CI, 30.0–70.0%) in the XFG group, with significantly worse outcomes in the XFG group (*p* = 0.03; Fig. 6). Table 5 shows the postoperative complications and interventions in the two groups. No significant differences were observed.

**Table 3:** The patient backgrounds of XFG and POAG matched using propensity score matching

	XFG (*n* = 24)	POAG (*n* = 24)	*p*-value	SMD
Male (eye)	13	9	0.39^#^	0.167
Age (years)	76.3 ± 6.0	77.0 ± 7.0	0.69^#^	0.107
Combined cataract surgery (eye)	1	0	1.00^*^	0.042
Preoperative IOP (mmHg)	20.7 ± 5.8	20.0 ± 7.6	0.72^#^	0.104
Preoperative number of medications (*n*)	4.4 ± 1.0	4.2 ± 1.1	0.57^#^	0.190

Data are shown mean ± standard deviation

*XFG* exfoliation glaucoma, *POAG* primary open-angle glaucoma, *IOP* intraocular pressure, *n*; number, *SMD* standardized mean difference (values < 0.2 indicate acceptable balance between groups)

^#^paired t test,*Fisher’s exact test

**Table 4:** Comparison of Postoperative Intraocular Pressure and number of medications Between of XFG and POAG matched using propensity score matching

	XFG (*n* = 24)	POAG (*n* = 24)	*p*-value*	XFG (*n* = 24)	POAG (*n* = 24)	*p*-value*
	IOP (mmHg)			Medication score (*n*)		
Pre operation	20.7 ± 5.8	20.0 ± 7.6	0.72	4.4 ± 1.0	4.2 ± 1.1	0.57
Post operation 1W	8.1 ± 3.5	8.4 ± 2.5	0.74	0 ± 0	0 ± 0	N/A
Post operation 1M	13.5 ± 5.4	11.2 ± 4.2	0.10	0.4 ± 1.2	0.1 ± 0.4	0.21
Post operation 3M	13.4 ± 5.4	13.7 ± 9.0	0.87	1.0 ± 1.7	0.4 ± 0.8	0.13
Post operation 6M	14.8 ± 6.1	11.9 ± 2.7	0.04	1.4 ± 1.8	0.5 ± 0.9	0.04

Data are shown mean ± standard deviation *paired *t*-test

*XFG* exfoliation glaucoma, *POAG* primary open-angle glaucoma, *IOP* intraocular pressure, *n* number, *W* week, *M* months, *N/A* not applicable

**Table 5.** Postoperative complications and interventions in XFG and POAG eyes

	XFG (*N*=24)	POAG (*N*=24)	*p*-value*
*Complications*			
Hyphema	8	3	0.17
Hypotony (≤ 5mmHg)	4	3	1.0
Choroidal detachment	2	1	1.0
Suprachoroidal hemorrhage	1	0	1.0
Vitreous hemorrhage	1	0	1.0
*Interventions*			
Needling	8	6	0.75
Additional surgery	4	1	0.35

*POAG* primary open-angle glaucoma, *XFG* exfoliation glaucoma, *Fisher’s exact test

## Discussion

Trabeculectomy has statistically superior IOP-lowering effects compared to PMS [33] and is indicated for cases that require a lower target IOP. However, in addition to the need for postoperative management, including laser suture lysis, the risk of vision loss due to postoperative hypotony makes it unsuitable for some glaucoma patients. By contrast, postoperative management of PMS—apart from the need for needling—is simpler than trabeculectomy because laser suture lysis is unnecessary. PMS is also less prone to intraoperative and postoperative hypotony, which may help preserve visual acuity after surgery.

This study evaluated the efficacy and safety of PMS in Japanese patients with XFG, a population considered at high risk for IOP management due to both their non-Caucasian ethnicity and the presence of XFG. PMS led to a significant reduction in IOP over 6 months, without severe vision-threatening adverse effects. Propensity score matching was used to compare the efficacy of PMS between XFG cases and a control group of POAG patients with similar baseline characteristics. Both groups showed significant reductions in IOP and medication scores; however, at 6 months, the IOP and medication scores were slightly higher in the XFG group than in the POAG group. The survival rate for IOP control at 6 months was also significantly lower in the XFG group than in the POAG group.

Several reports have examined the efficacy and safety of PMS for eyes with XFG. Antonio et al. conducted a multicenter study in which PMS with MMC concentrations of 0.02–0.05% was performed on 81 eyes with POAG and 23 with XFG [17]. At 12 months postoperatively, IOP had decreased from 25.0 ± 6.7 to 14.3 ± 3.6 mmHg in POAG eyes, and the number of glaucoma medications had decreased from 2.9 ± 1.0 to 0.8 ± 0.9. In comparison, in XFG eyes, IOP had decreased from 25.0 ± 5.9 to 13.5 ± 2.4 mmHg and the number of medications had decreased from 3.0 ± 1.0 to 0.8 ± 1.0. The IOP reduction (survival rate) at 12 months did not differ significantly between the two groups (*p* = 0.52), and no difference was observed between phakic and pseudophakic eyes. In another report using a 0.02% MMC concentration, Nobl et al. inserted the PMS in 20 XFG and 26 POAG eyes. At 12 months postoperatively, no significant difference in IOP or medication use was found between the two groups [28]. The survival rate (IOP reduction of > 20% from baseline and within 5–16 mmHg) also did not differ significantly between the groups. Regarding complications, XFG patients had higher incidences of anterior chamber hemorrhage, hypotony, and choroidal detachment than POAG patients. A report from Japan by Wakuda et al. describes PMS with 0.04% MMC in 29 eyes of Japanese patients with XFG [29]. At 46 weeks postoperatively, IOP had decreased from 32.6 ± 9.1 to 16.9 ± 10.5 mmHg, and the number of glaucoma medications had decreased from 3.4 ± 1.0 to 1.0 ± 1.3. In their study, which evaluated IOP management under relatively similar conditions (success defined as a ≥20% reduction in IOP, with 5–15 mmHg), the complete success rate was 49%, and the conditional success rate was 53%, comparable to our findings. Table 6 summarizes a comparison between the results of previous studies and those of the present study.

**Table 6:** Comparison of previous studies reporting outcomes of PreserFlo MicroShunt in patients with XFG and POAG

	Antonio et al. [17]		Nobl et al. [28]		Wakuda et al. [29]		Present study
Country	Italy, Sweden, and United Kingdom		Germany		Japan		Japan
MMC concentration	0.02–0.05%		0.02%		0.04%		0.04-0.05%
Follow-up duration	12 months		12 months		6 months		6 months
Diagnosis	XFG	POAG	XFG	POAG	XFG	XFG	POAG
Eyes (n)	23	81	20	26	29	24	24
Preoperative IOP (mmHg)	25.0 ± 5.9	25.0 ± 6.7	21.4 ± 5.8	18.2 ± 4.5	32.6 ± 9.1	20.7 ± 5.8	20.0 ± 7.6
Postoperative IOP (mmHg)	13.5 ± 2.4	14.3 ± 3.6	12.8 ± 3.0	12.9 ± 4.2	16.9 ± 10.5	14.8 ± 6.1	11.9 ± 2.7
Preoperative number of medications (n)	3.0 ± 1.0	2.9 ± 1.0	2.8 ± 1.3	2.7 ± 1.3	3.4 ± 1.0	4.4 ± 1.0	4.2 ± 1.1
Postoperative number of medications (n)	0.8 ± 1.0	0.8 ± 0.9	0.3 ± 0.8	0.3 ± 0.8	1.0 ± 1.3	1.4 ± 1.8	0.5 ± 0.9

Data are shown mean ± standard deviation

*MMC* mitomycin C, *XFG* exfoliation glaucoma, *POAG* primary open-angle glaucoma, *IOP* intraocular pressure, *n* number

Previously reported values are cited from Antonio et al. [17], Nobl et al. [28], and Wakuda et al. [29]

Our study and the aforementioned reports from abroad differ in terms of the target population, making direct comparisons challenging, particularly given that the results are being compared at 6 months postoperatively. The mean IOP and number of glaucoma medications after PMS surgery for XFG in our study were slightly higher than those reported internationally. This discrepancy may be attributed to the Asian race, a potential risk factor for intraocular pressure management [34]. In contrast, a study by Wakuda et al. on Japanese XFG patients [29]—similar to those in our study—reports a higher preoperative mean IOP (32.5 mmHg). Additionally, their study included cases in which the tube was inserted in the lower quadrant. Despite these differences, the success rate was comparable to our results, with a success rate of 49–67% at 24 weeks. Further investigation is needed to better understand postoperative IOP dynamics in Japanese XFG patients.

The rate of additional surgery was 10% in the study by Nobl et al., 31% in Wakuda et al., and 16% in our study. Since the criteria for additional surgery vary across institutions, it is difficult to determine the significance of these differences. In our propensity score-matched comparison with POAG, no significant difference was observed in the rate of additional surgery.

The novelty of this study lies in the use of propensity score matching to establish a control group (POAG group) with baseline characteristics that matched those of the XFG group, enabling a direct comparison of postoperative outcomes between the two glaucoma types. At 6 months postoperatively, IOP and glaucoma medication scores were significantly higher in the XFG group than in the POAG group. The qualified success rate was also higher in the POAG group. So far, studies that have compared XFG and POAG in PMS are limited; although the follow-up period was short (one year), one study reports that micro-shunts demonstrated similar efficacy in XFG and POAG, despite a high incidence of transient hypotony and choroidal detachment [28].

On the other hand, previous reports indicate that the success rate of trabeculectomy for XFG is lower than for POAG [8, 9]. This difference is thought to arise from the distinct pathophysiological characteristics of XFG. After trabeculectomy in XFG, disruption of the blood-aqueous barrier, inflammatory responses, and fibrin exudation are thought to accelerate filtering bleb scarring and reduce its filtering capacity [10, 35]. The higher risk of anterior chamber inflammation and the greater tendency for scar tissue formation in XFG further contribute to its lower success rate. On the contrary, the PMS is thought to cause less tissue invasion and weaker inflammatory responses than trabeculectomy; these factors may still lead to more pronounced filtering bleb scarring in XFG than in POAG. Sugimoto et al. report that in eyes with XFG undergoing Ex-PRESS surgery, the volume of the fluid-filled space within the filtering bleb was significantly smaller at 3 and 6 months postoperatively compared to eyes with POAG [36]. This finding suggests a greater propensity for scar tissue formation in the bleb during the 3–6-month postoperative period in XFG eyes, which may account for the higher intraocular pressure observed at 6 months in XFG eyes compared to those with POAG in the present study. As the follow-up period extends, the impact of this scarring would become more evident, making long-term IOP control increasingly challenging.

Postoperative IOP values in XFG patients may be slightly higher than in POAG patients; however, PMS surgery remains a viable option due to its potential to suppress postoperative IOP fluctuations. Studies indicate that IOP fluctuation is greater in XFG than in POAG [4] and this is thought to affect glaucoma progression negatively. In cases where progression occurs despite normal IOP management, both short- and long-term IOP fluctuations are thought to contribute to disease progression [37–39]. If the PMS can effectively stabilize IOP fluctuations, it may play a significant role in slowing disease progression. One study suggests that the PMS can reduce IOP fluctuations [40], but further investigations in larger patient cohorts are necessary to confirm this.

Marta et al. report a 5.1% decrease in CECD at 6 months following PMS implantation [41]. In their study, 43 of 46 eyes were diagnosed with POAG, and 2 eyes with XFG. In the present study, no significant decrease in CECD was observed at 6 months postoperatively; however, a reduction exceeding 5% was noted in 11 of 31 eyes (35.5%). Consistent with previous reports, which predominantly involved eyes with POAG, this study also found no significant CECD decrease at 6 months postoperatively in eyes with XFG. Nevertheless, long-term follow-up is necessary to ascertain whether this trend persists and to evaluate the potential cumulative effects of the procedure on corneal endothelial cell density.

This study has several limitations that should be considered when interpreting the results. First, as a retrospective cohort study, it was impossible to eliminate all known and unknown confounding factors or biases. To mitigate this, relatively strict inclusion and exclusion criteria were applied; however, complete control of confounders was not achievable. Additionally, patients who were transferred to other institutions during the follow-up period were excluded from the analysis, potentially limiting the generalizability of the findings. Furthermore, the criteria for repeat surgery, needling, and resumption of glaucoma medications were not standardized, and their potential influence on the outcomes must be considered. Postoperative steroid tapering protocols may also have varied between XFG and POAG patients because of the experience that XFG is more likely to cause conjunctival scarring, potentially affecting IOP management. However, it is unclear at which postoperative stage the difference arises, due to the small number of measurement points, it cannot be clearly stated. Thus, extended observation is necessary to fully understand the postoperative management required for XFG. A comparison of the efficacy and safety of PMS and trabeculectomy in XFG eyes remains an issue to investigate. In a cumulative case series performed by a single surgeon at a single institution, the six-month outcomes of trabeculectomy in XFG eyes (mean age: 74.4 years, baseline IOP: 23.1 mmHg) showed a mean IOP of 12.9 mmHg and a cumulative survival rate (IOP < 15 mmHg) of 78.3% [42]. Although direct comparison is difficult due to differences in patient backgrounds, our PMS results show higher IOP and lower survival rates at six months postoperatively compared to their report. The limited sample size and involvement of multiple surgeons may have contributed to this outcome, but PMS may have inferior results compared to trabeculectomy. Another limitation is the lack of a clear consensus on the optimal MMC concentration for glaucoma surgery. In this study, a concentration of 0.04–0.05% was used in both the XFG and POAG groups, which is slightly higher than the concentrations commonly used in other countries. This concentration represents the standard protocol adopted at our institution for both POAG and XFG cases [26]. Given these limitations, caution is needed when generalizing the findings of this study. Finally, when evaluating CECD, gonioscopic evaluation was not performed in all cases, and detailed anterior segment imaging using OCT was also not performed, making it difficult to interpret the cause of the decrease in CECD. To overcome these limitations, future multicenter collaborative studies and prospective research are warranted to address these limitations and strengthen the evidence base.

In conclusion, this study demonstrates that the PMS significantly reduced IOP and decreased glaucoma medication scores in the eyes of Japanese patients with XFG at 6 months postoperatively. Notably, the improvement in IOP control and reduction in the burden of glaucoma medications implies that this treatment enhances patients’ quality of life. Regarding safety, although some transient adverse events were observed, no severe long-term complications, such as vision loss, were reported, supporting the overall safety of this procedure. However, compared to POAG, treatment outcomes appeared less favorable in XFG eyes. These findings provide preliminary evidence supporting the usefulness of the PMS as a treatment option for Japanese XFG patients. However, further research is warranted to confirm its long-term efficacy and safety.
